# Nanoengineering of conductively coupled metallic nanoparticles towards selective resonance modes within the near-infrared regime

**DOI:** 10.1038/s41598-022-11539-4

**Published:** 2022-05-12

**Authors:** Naby Hadilou, Somayeh Souri, H. A. Navid, Rasoul Sadighi Bonabi, Abbas Anvari

**Affiliations:** 1 Department of Physics, Sharif University of Technology, Tehran, Iran; 2 Department of Laser and Optical Engineering, University of Bonab, Bonab, Iran

**Keywords:** Mathematics and computing, Nanoscience and technology, Optics and photonics

## Abstract

In this work, the mode transition effect of different plasmonic resonances in linked dimers by a conductive junction is numerically investigated.Without the junction, the dimer supports a single dipolar bonding plasmon mode, while two new resonance modes, a screened bonding dipolar mode and a low energy charge transfer plasmon mode, emerge when two nanoparticles are linked via a bridge. Such effect is proved to be unrelated to the shape of the nanoparticles, whether sphere, core-shell or nanoegg. However, it was found that the status of each specific resonance mode is profoundly influenced by the shape of nanoparticles. Furthermore, a detailed discussion of mechanisms of controlling plasmon modes, specially charge transfer mode, and tuning their corresponding spectra in bridged nanoparticles as functions of nanoparticle parameters and junction conductance is presented. These results show that the optical response of the dimer is highly sensitive to changes in the inter-particle gap. While the capacitive dimer provides a strong hotstop, the conductive dimer leads to highly controllable low energy plasmon mode at the mid-infrared region appropriate for novel applications. These findings may serve as an important guide for optical properties of linked nanoparticles as well as understanding the transition between the capacitive and conductive coupling.

## Introduction

Over the past two decades, there is a great interest in the development of metallic nanostructures due to their fascinating optical properties dominated by their Localized Surface Plasmon Resonance (LSPR) which is the collective oscillation of conduction electrons in response to an external electromagnetic field. The frequency, strength and quality of LSPR of the single nanoparticle can be tuned by changing the nanoparticle’s size, geometry, material and the surrounding medium^1^. Moreover, the presence of the other nearby metal nanoparticle strongly affects LSPR and the optical response of the nanostructure^2,3^.

When two nanoparticles are placed next to each other with an inter-particle gap smaller than the diameter of the individual nanoparticles to form a dimer, the properties of their surface plasmons are significantly modified due to the interaction and hybridization of plasmon modes of individual nanoparticles, resulting in new hybridized dimer plasmon modes^4–6^. The spectra of dimers differ from those of single nanoparticles. In particular, energies of dimer plasmon modes experience a redshift relative to individual plasmon modes and electromagnetic fields are significantly localized and enhanced in the inter-particle gap known as a hotspot due to a strong capacitive coupling of plasmon modes^4,7,8^. Owing to the giant E-field enhancement within the hotspot, dimeric nanostructures contribute a major role in several practical applications such as surface enhanced Raman scattering (SERS)^9^, molecule detection^10^, nanoantenna^11^ and photovoltaic cell design^12^.

The electromagnetic field enhancement in the hotspot can be profoundly modified by structural design^13–15^. Besides the length of the inter-particle gap of the dimer, one of the most dramatic modifications occurs when the geometries of individual nanoparticles change^16–20^. Among numerous shapes, dimer composed of metal nanospheres is the most popular due to its easy fabrication process. However, using core-shell structures to comprise the dimer provide much wider spectral tunability, as well as further electric field enhancement^21–23^. The energy of LSPR and strength of the hotspot can be easily tuned by varying the core to shell radii ratio. Moreover, giving the core an offset, forming an asymmetric configuration called nanoegg, offers new possibilities for tailoring the spectra and amplifying the amplitude of the electric field within the gap^24–26^.

The conductivity of the medium filled the inter-particle gap is another critical parameter in plasmonic coupling. Generally, the plasmon modes of two nanoparticles would be coupled conductively or capacitively^27–32^. Recent reports reveal that a transition from capacitive to conductive coupling by filling the gap with a conductive junction, forming a bridged dimer, leads to a tremendous modulation of the spectral feature of the dimeric configuration^33–36^. In capacitive coupling, where the gap is filled with a capacitive material such as air, the optical properties of the nanostructure are dominated by bonding dimer plasmon (BDP) mode. i.e, the dipolar modes of individual nanoparticles hybridize and localize the charge density on each side of the gap. In conductive coupling, in contrast, the charges are allowed to oscillate between nanoparticles through the conductive path, resulting in a new resonance mode in the optical spectra of the nanostructure known as a charge transfer plasmon (CTP) mode. On the other side, when the surface charges on the opposite sides of the junction have had time to neutralize, another resonance mode called screened bonding plasmon (SBDP) mode appears in the spectra^35^. While SBDP mode occurs at higher energies, CTP is a low-energy plasmon mode that jumps up to the infrared regime. The CTP modes possess attractive features across the mid-infrared domain with high tunability by manipulating the junction profile and nanoparticles geometries. Realizing these features across low energies would allow for novel applications such as molecular sensing and nano-optoelectronic device^29,37–39^. Understanding CTP properties can help researchers to achieve highly tunable plasmon resonances into the infrared region of the spectrum. In addition, since the intrinsic of CTP is related to the charge flow, having knowledge of linked nanoparticles can be helpful for studying the electron transport of molecules at optical frequencies that are not accessible electronically^37^.

In this paper, the optical properties of both capacitive and conductive coupled nanoparticles (i.e., dimer and bridged dimer) are numerically investigated by 3D full electrodynamics finite element method simulations. Here, we seek an understanding of how plugging a bridge between adjacent nanoparticles dramatically changes the excitation energy of the plasmon resonance as well as the distribution of electric field within the inter-particle gap. In addition, three dimeric nanostructures composed of two nearby spheres, core-shells and nanoeggs are considered to elucidate the impact of the geometry and breaking symmetry on the optical response of nanostructures. Moreover, tuning the intensity and resonance energy of charge transfer plasmon mode in the linked dimers by varying the width and length of the junction is precisely studied.

## Model and methods

Here, the coupling of electromagnetic waves with the dimeric nanostructures is numerically investigated by solving Maxwell’s equations in the classical regime using the local dielectric function for metallic material and connecting nearby nanoparticles by a conductive junction to consider the effect of current tunneling. Both the optical response and the near field enhancement of plasmonic nanostructures including dimer and bridged dimer will be calculated by performing 3D modeling and simulation based on the finite element method (FEM) using a software package COMSOL Multiphysics (version 5.3a). In particular, the radio frequency module was employed to characterize the optical properties of these dimeric nanosystems.

Before going into the details of conductively coupled dimers and the effect of the junction on the spectra, to have a grasp of the spectral evolution, the properties of capacitively coupled dimers are also investigated. Therefore, two model systems of dimers and bridged dimers are considered to make a complete comparison. In each system, the individual nanoparticles are in three shapes: sphere, core-shell and nanoegg. The geometry of dimers is defined by three parameters, core $(R_c)$ and shell $(R_s)$ radii of individual nanoparticles and interparticle-gap (*g*). For nanoegg, the inner core is moved away from the center $(\sigma )$ but does not touch the shell. The dimeric nanostructures composed of two spheres, core-shells and nanoeggs are abbreviately called SSD, CSD and NED, respectively. For bridged dimer, besides the geometric parameters of adjacent nanoparticles, the geometry and conductance of the junction must be clarified. It is considered that two nanoparticles are linked by a cylindrical conductor of radius *a* and length *L*. The conductance *G* of the bridge is defined by the conductivity $\sigma _J$ and geometric parameters including the length of the bridge and the cross-section of the contact area (*A*) as follow:^33^

1 $$\begin{aligned} G=\sigma _J\frac{A}{L}. \end{aligned}$$

**Figure 1:**
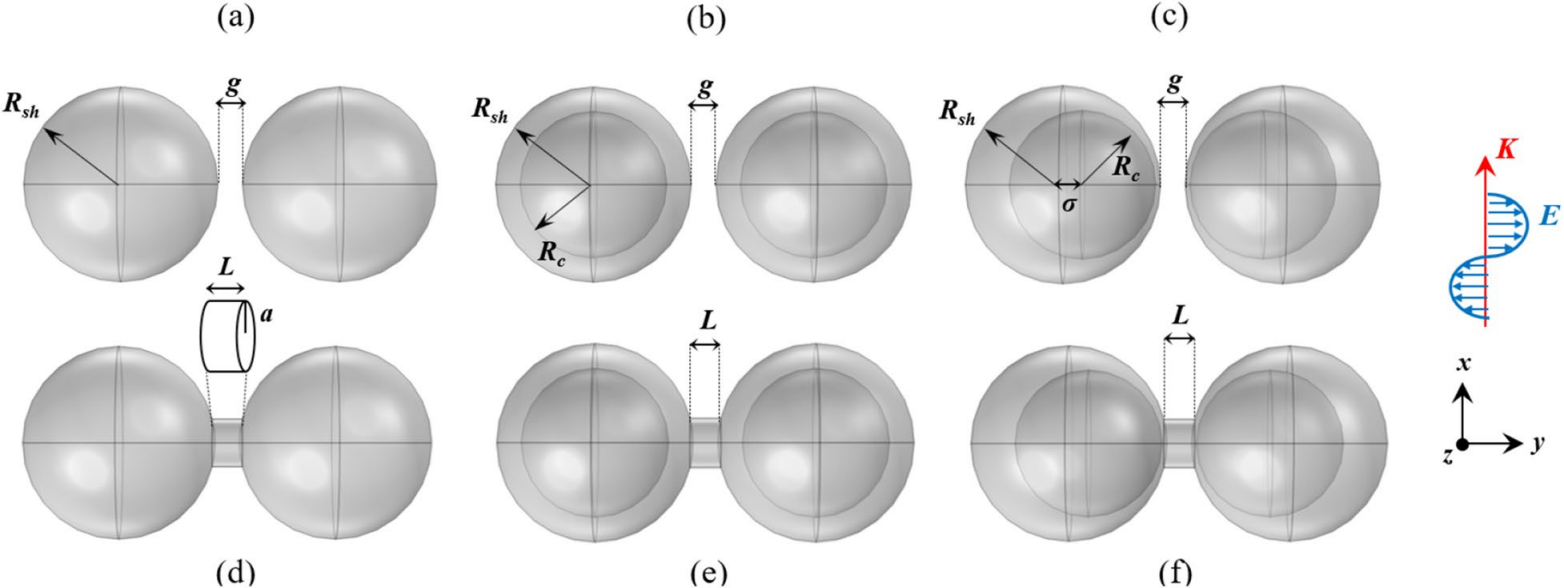
Schematic diagram of (top) dimers and (bottom) bridged dimers with different geometries including (**a**) spherical dimer—SSD, (**b**) core-shell dimer—CSD, (**c**) nanoegg dimer—NED, (**d**) spherical bridged dimer—SSBD, (**e**) core-shell bridged dimer—CSBD and (**f**) nanoegg bridged dimer—NEBD.

Similarly, the abbreviations of SSBD, CSBD and NEBD are defined for the bridged dimers comprised of two spheres, core-shells and nanoeggs for simplicity. The model systems under study are depicted in Fig. 1. It is worth mentioning that these nanostructures are proposed based on the recent nanofabrication techniques^33,37,40–42^.

Gold in nanoscale form exhibits the most interesting optical properties as well as selective LSPR in the visible and near-infrared regime. Therefore, the optical properties of dimer in which the metal of choice is gold are investigated. In the case of core-shell and nanoegg, the core is made of silica $(\varepsilon _{SiO_2}=2.04)$^43^ and the nanosystems are assumed to be suspended in vacuum $(\varepsilon _m=1)$. The frequency-dependent dielectric function of gold is given by the Drude–Lorentz model:^44^

2 $$\begin{aligned} \varepsilon (\omega )=\varepsilon _{\infty }+\frac{\omega _p^2}{\omega ^2+i\omega \gamma _{bulk}} -\frac{\omega _p^2}{\omega ^2+i\omega (\gamma _{bulk}+\gamma )}. \end{aligned}$$

$\varepsilon _{\infty }$ is the bulk dielectric function for gold, $\omega _P=9eV$ is the plasma frequency, $\omega$ is the photon energy and $\gamma _{bulk} =0.066$ eV is the electron collision damping in the gold. For nanoparticles interband transition must be considered by $\gamma = Av_f/L{eff}$, where A is a dimensionless fitting parameter for matching theoretical and empirical data and is taken as unity in all simulations, $v_f$ is the Fermi velocity $(v_f=1.4 \times 10^6)$ m/s and $L_{eff}$ is the effective mean free path of electron and is taken to equal to the radius of nanoparticle^45^.

A plane wave illuminates the nanostructures at normal incident along the z-axis (perpendicular to the dimer axis). The polarization of the electric field is along a dimer axis (x-axis) with the background electric field of $E_b=E_0exp(-ikz)$, where $E_0$ is the incident electric field, which is set to unity in this numerical study and $k=\frac{2\pi }{\lambda }\sqrt{\varepsilon _m}$ is the wavenumber of light in the surrounding medium. Note that the electric field distribution is computed using Helmholtz equation^46^.

The optical response of nanostructures is explored in terms of their extinction efficiency which is a sum of scattering and absorption abilities. The scattering and absorption and cross-sections are calculated as follow:

3 $$\begin{aligned} \sigma _{sca}= & {} \frac{1}{I_0} \int \int \mathbf{S _{sca} \cdot \mathbf{n} }dS \end{aligned}$$

4 $$\begin{aligned} \sigma _{abs}= & {} \frac{1}{I_0} \int \int \int {Q_hdV}, \end{aligned}$$

where $\mathbf{S} _{sca}$ and $Q_h$ are the Poynting vector and the total power dissipation density, respectively. $\mathbf{n}$ is the normal vector pointing outward from the surface of the nanostructures. *S* and *V* represent the surface and volume of the nanostructures, respectively. $I_0=|\frac{1}{2}\varepsilon _0 nE_0^2|$, is the incident energy flux, where *n* is a refractive index of surrounding medium. The extinction cross-section is calculated as $\sigma _{ext} = \sigma _{sca}+\sigma _{abs}$. These equations are solved in a domain of radius corresponding to the irradiation wavelength. Perfectly Matched Layers (PMLs) are used to ensure total absorption of the electromagnetic radiation at the simulation boundaries. Thickness of PML for surrounding medium was selected suitably to incident wavelength. Discretization of the simulation space was performed using the built-in free meshing COMSOL algorithm, with choosing division into “finer” tetrahedron elements.

Another important parameter that must be evaluated to confirm the nature of resonance plasmon mode is the induced charges on the nanoparticle surface, $Q=\int \int \rho dS$, where $\rho$ is the surface charge density:^47–49^

5 $$\begin{aligned} \rho =\varepsilon _0 \cdot (\mathbf{n} \cdot {{\varvec{E}}}) =\varepsilon _0 (n_x \cdot E_x + n_y \cdot E_y+n_z \cdot E_z) \end{aligned}$$

## Results and discussion

### The role of nanoparticles

To understand the effect of conductivity of the medium in the gap region on the optical response of coupled nanoparticles, two different nanostructures of dimer and bridged dimer are explored. The discussion is initiated by investigation of the effect of geometry on plasmonic properties and local hotspots of dimers composed of two identical spheres, core-shells and nanoeggs (i.e., SSD, CSD and NED). Then, it continues by inserting the bridge into the gap to link the nanoparticles (i.e., SSBD, CSBD and NEBD) for studying the huge spectral changes due to the transition from capacitive to conductive coupling.

**Figure 2:**
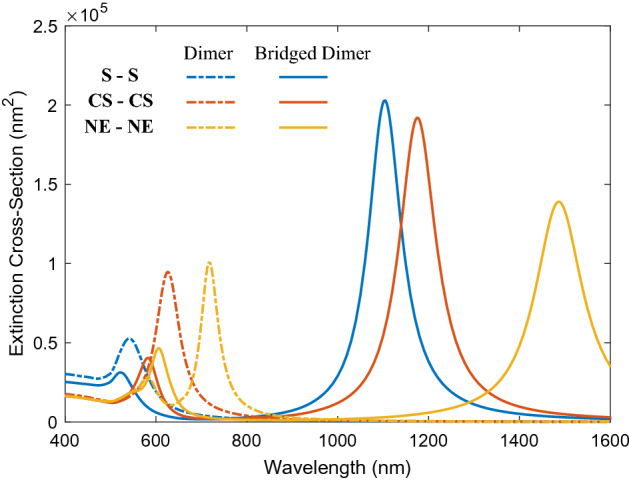
Extinction spectra of dimers and bridged dimers with different geometries. The dashed lines display the extinction cross-sections of bonding dimer plasmons of the dimers having 10 nm gap. The solid curves show the extinction cross-sections of screened bonding modes and charge transfer modes of conductively linked nanoparticles. The radius and length of the bridge are 10 nm. In all cases the radius of the shell is 40 nm. For nanostructures with cores and offset, the radius of the core is 30 nm and the core is replaced by 8 nm.

We will consider the dimers composed of two spherical nanoparticles of radii 40 nm separated by a 10 nm gap. For the symmetric and asymmetric core-shells, the radii of the core are set to 30 nm and the core offset is 8 nm for NED and NEBD. In the bridged dimer, the gap is filled with the conductive cylindrical shape junction of radius 10 nm and length of 10 nm. Note that these values are specific to this section, while a range of values for the core radius and offset as well as the radius and length of the bridge are considered to evaluate their impact on the optical behaviors. The conductivity of the bridge is similar to the conductivity of metal particles. It is possible to modify $\sigma _J$ of nanolinker, however, we use the junction with the same metallic character since the CTP becomes prominent in the spectrum at this condition. In all simulations, the size of dimers and bridge are chosen such that the classical approach is valid. Therefore, the minimum separation is set to 2 nm to avoid nonlocality and quantum effects^50,51^. Note that nanoeggs can place next to each other in different ways depending on the direction of their offsets. In this part, we only consider the orientation as the cores move toward the bridge (see Fig. 1). The extinction spectra of capacitive and conductive coupled nanoparticles in dimers are shown in Fig. 2.

**Figure 3:**
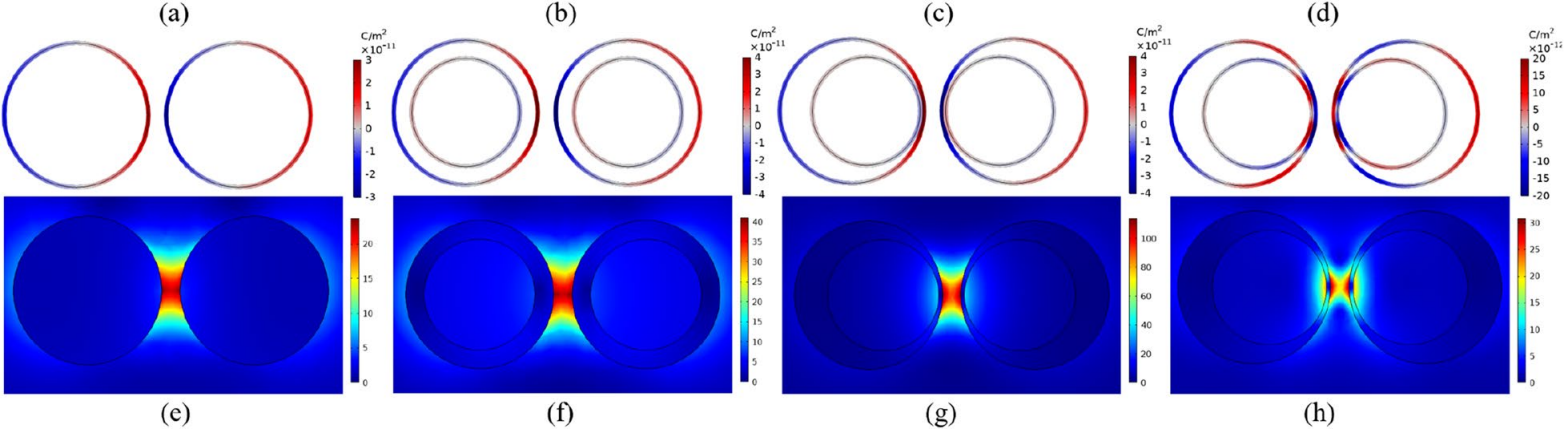
Top panel: Surface Charge distribution of bonding plasmon mode of (**a**) SSD (**b**) CSD, (**c**) NED and (**d**) higher-order plasmon mode of NED. Red color represents positive charge while blue is negative. Bottom panel: E-field maps of the dimer calculated at the resonance wavelength. (**e**) SSD at $\lambda _{\text {BDP}}=542\;\text {nm}$, (**f**) CSD at $\lambda _{\text {BPD}}=625\; \text {nm}$, (**g**) NED at $\lambda _{\text {BDP}}=719 \; \text {nm}$ and (**h**) NED at $\lambda _{\text {HO}}=584 \; \text {nm}$.

As one can see, when the gap is filled with air (dashed lines), the spectrum is dominated by a pronounced peak of BDP in the visible region due to the strong interaction of dipole modes of primitive nanoparticles. It is also known as gap plasmon resonance (GPR)^52^. For NED, the interaction of quadrupole and dipole modes of each nanoeggs is allowed due to the symmetry breaking, resulting in an additional resonance mode at the blue side of the original peak. In order to visualize the nature of the plasmonic modes, the surface charge density of each mode is plotted in the top panel of Fig. 3. This mapping illustrates the features of dipole and quadrupole modes. From Fig. 3a–c, for SSD, CSD and NED irradiated at 542 nm, 625 nm and 719 nm, respectively, there are two induced charges poles in the spatial distribution of surface charge density corresponding to fundamental dipole modes. However, the resonance peak at 584 nm of NED is dominated by the quadrupolar plasmon resonance since there are three induced charge poles (Fig. 3d).

**Figure 4:**
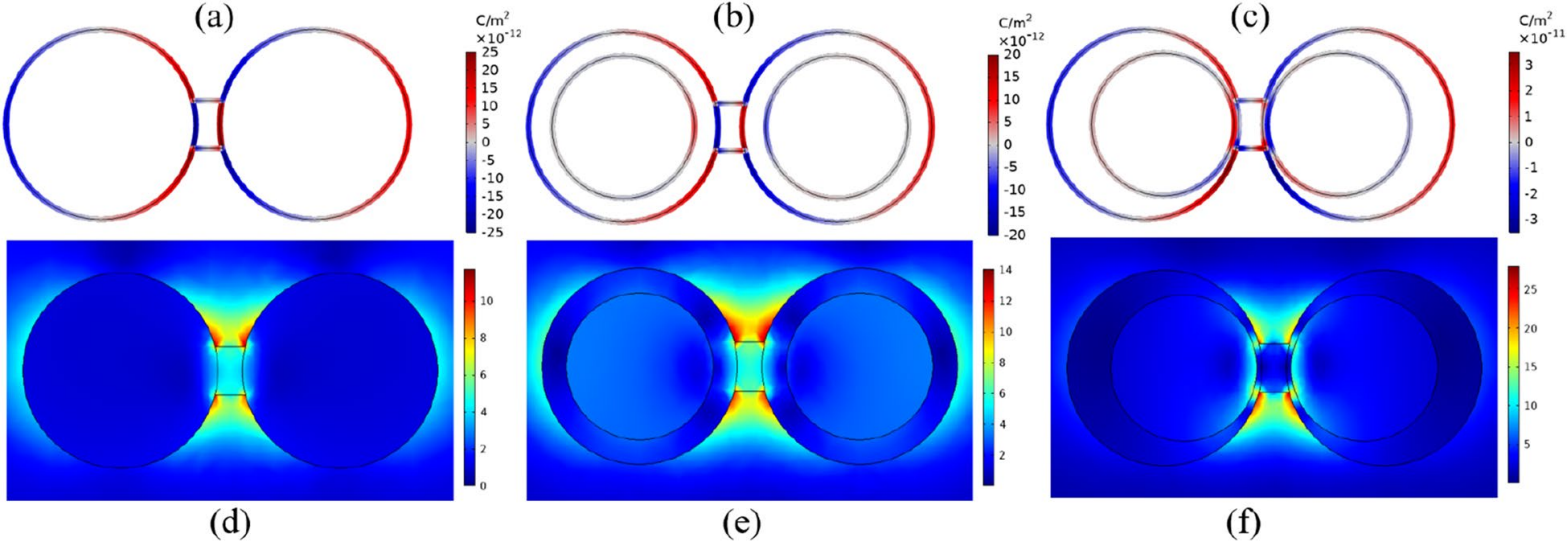
Top panel: Surface Charge distribution of SBDP mode of (**a**) SSBD (**b**) CSBD, (**c**) NEBD. Red color represents positive charge while blue is negative. Bottom panel: E-field maps of the bridged dimer calculated at SBDP. (**d**) SSBD at $\lambda _{\text {SBDP}}=522 \; \text {nm}$, (**e**) CSBD at $\lambda _{\text {SBDP}}=581 \; \text {nm}$ and (**f**) NEBD at $\lambda _{\text {SBDP}}=605 \; \text {nm}$.

**Figure 5:**
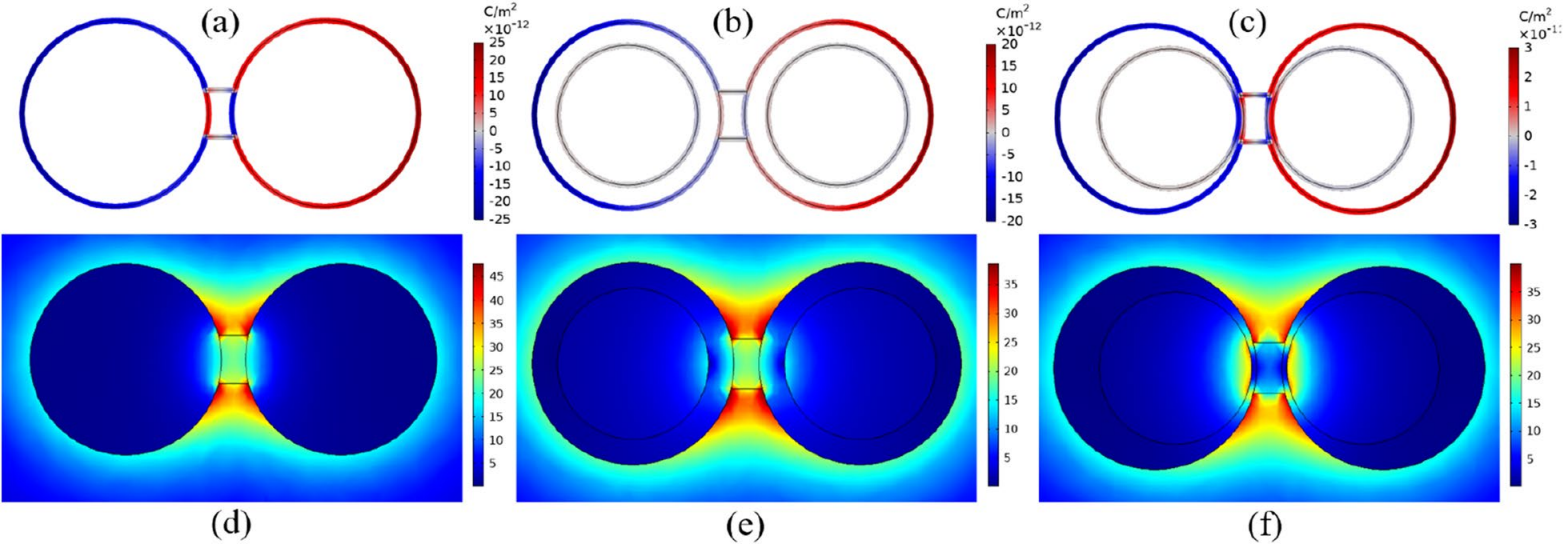
Top panel: Surface Charge distribution of CTP mode of (**a**) SSBD (**b**) CSBD, (**c**) NEBD. Red color represents positive charge while blue is negative. Bottom panel: E-field maps of the bridged dimer calculated at CTP. (**d**) SSBD at $\lambda _{\text {CTP}}=1104 \; \text {nm}$, (**e**) CSBD at $\lambda _{\text {CTP}}=1176 \; \text {nm}$ and (**f**) NEBD at $\lambda _{\text {CTP}}=1487 \; \text {nm}$.

From Fig.  2, it is clear that the BDP of CSD and NED is significantly redshifted compared to the dipolar mode of SSD. This can be interpreted as follows: firstly, we investigate the difference in the optical response of individual nanoparticles with three different geometries (i.e., solid, core-shell and nanoegg). In contrary to spherical solid NP, nanoshell possesses two tunable resonances arising from the hybridization of the plasmons on the inner surface of the shell (cavity mode) and the plasmons on the outer surface of the shell (spheroid mode). These two plasmon resonances of a nanoshell which are redshifted and blueshifted relative to the plasmon mode of spherical NP are denoted by the bonding and antibonding plasmonic modes, respectively^53,54^. It is worth emphasizing that the antibonding mode resonance is hardly excited by the illumination of a plane wave^55^. Therefore, one pronounces redshifted resonance peak corresponding to bonding plasmon mode of nanoshell is a signature of its optical spectrum. It is well known that the displacement of the resonance mode toward the larger wavelengths is determined by the strength of hybridization. The stronger is the hybridization, the larger is the redshift of the LSPR peak. Since the strength of hybridization is highly geometrically sensitive, introducing an offset to the core significantly affects the position of bonding plasmon mode of nanoshell in the spectrum due to a variation of the parental plasmon modes energies, resulting in a redshift of bonding plasmon mode. In other words, as the core is offsetting, the hybridization between the cavity and spheroid modes becomes progressively stronger due to the change in the charge distribution on the inner and outer surfaces of the nanoshell, leading to a larger redshift of bonding mode^45^. Now with this insight, we can explain the reason for the larger redshift of CSD and NED in comparison with SSD. It is shown that the plasmon modes of individual nanoparticles with different shapes of sphere, core-shell and nanoegg have different energies (wavelengths). When these NPs bring close to each other, their bonding plasmon modes couple and create the gap plasmon mode of the dimer (i.e., BDP). The BDP shift in the spectrum depends on the energies of the two hybridizing modes. Therefore, it is expected that the hybridization of highly redshifted bonding modes of NEs leads to a larger redshift of BDP of NED compared to two other structures. Furthermore, in symmetric core-shell, only the parental modes with the same order can interact with each other, while the modes with different order can be hybridized in asymmetric nanoegg. This is why another peak is observed in the spectrum of dimers composed of two nanoeggs.

Moreover, changing the geometry of dimeric nanostructure strongly affects the electric field strength in the hotspot region. The bottom panel of Fig. 3 shows the local electric field distributions calculated at the resonance wavelengths. For all nanostructures, strong capacitive coupling of nanoparticles leads to an intense E-field in the nanogap regions. However, the strength of the hotspot is notably enhanced by changing the geometry. The coupled nanoeggs achieve the strongest localized E-field in the gap at BDP, followed by the coupled core-shells and spheres. This substantial enhancement for NED makes them more attractive in practical applications.

A further step in our study is filling the nanogap with the conductive medium by considering a bridge between two nanoparticles. Figure 2 shows how the extinction spectra evolve as the dimer is connected through a conductive bridge. When two nanoparticles are connected by a conductive bridge, they are coupled both capacitively and conductively. The BDP mode suddenly splits into two discrete modes; a high-energy resonance mode called screened bonding plasmon (SBDP) mode and a new low-energy resonance mode called charged transfer plasmon (CTP) mode. The former is recognized as a blueshifted bonding mode due to a weaker polarization of the charge distribution of the individual nanoparticles and the latter is ascribed to an oscillating electrical current across the junction. In other words, once the dimer is connected, the charge can flow between nanoparticles giving rise to the charge transfer plasmon mode in the long-wavelength region (1000–1600 nm). Simultaneously, by decreasing the capacitive coupling, the energy of BDP tends to blueshift and forms a screened bonding plasmon mode due to the screening effect of the bridge on coupling^36,52,56,57^. For SBDP, the local electric field is still present across the bridge, allowing for capacitive coupling between the two nanoparticles. In this case, the capacitive coupling is reduced, resulting in a slight blueshift of the BDP. However, the electric field is expelled from the junction for CTP. In this limit, the bridge acts as a short circuit allowing a charge transfer between nanoparticles^35^. This fact is observed for all nanostructures and such an effect is unrelated to the shape of the nanoparticles. However, the wavelengths of resonance modes as well as their extinction efficiencies are different. The position of low-energy CTP modes of SSBD, CSBD, and NEBD is 1104 nm, 1176 nm, and 1487 nm, and their SBDP modes are located at 522 nm, 581 nm, and 605 nm, respectively.

Both the CTP and SBDP modes involve the oscillatory charge transport across the bridge. As mentioned, mapping the distribution of the surface charge density and the electric field is the best way to illustrate the nature of plasmon modes. As illustrated in the top panel of Fig. 4, the pole near the gap is deformed and reduced in magnitude. Although the charge appears on the edge of the bridge, the dipolar modes of nanoparticles are still visible. Compared to the dimer, the presence of the bridge shifts the resonance to the blue region and dramatically decreases the field enhancement (bottom panel of Fig. 4). The reason can be interpreted as follow: In the absence of the bridge, there is a large Coulomb attraction between two oppositely signed sides of nanoparticles (BDP). However, the surface charges facing the junction are shorted by inserting the bridge, resulting in reducing the Coulomb attraction. In other words, reducing the capacitive coupling by screening the charge transfer, induce a blueshift of resonance and a weaker electric field within the gap.

The CTP, as shown in the top panel of Fig. 5, is formed when the charge flows from one nanoparticle to the other, and, one nanoparticle becomes a positive pole and the other a negative pole. Therefore, CTP modes are sometimes considered as a hybridization of monopole modes^56^. Furthermore, two connected nanoparticles can be considered as a continuous particle of longer length, in which the fundamental dipole mode is excited. It is well-known that the larger is the nanoparticle, the larger is the redshift of the plasmon mode. Consequently, the CTP experiences the redshift toward the mid-infrared region. The bottom pannel of Fig. 5 shows the local electric field distribution for the CTP modes. Still, enhanced E-field distribution around the bridge, however, no electric field is observed inside the bridge which acts as a short circuit.

The CTP is a highly tunable mode that its energy and intensity can be modified by controlling the geometries of the nanoparticles and the junction. Figure 2 shows the effect of nanoparticles shape on the tunability of CTP spectral peaks. The extinction spectra of bridged dimer of various shapes reveal that the CTP exhibits a remarkable redshift from 1104 to 1176 nm and 1483 nm with changing the shape of nanoparticles from sphere to core-shell and nanoegg, respectively, while the magnitude of extinction cross-sections monotonically decreases. Similarly, the shape of nanoparticles affects the wavelength of SBDP, moving from 522 nm in SSBD to 581 nm in CSBD and 605 nm in NEBD. However, the magnitude of extinction cross-section of SBDP increases.

The significant redshift of CTP can be interpreted in terms of the time needed for the electrons to cross the junction which is defined as the ratio of transferred charges to the current in the junction. The current is proportional to the local electric field $E^{\text {loc}}$ inside the bridge $(J\propto GLE^{\text {loc}})$^35,58^. In this case, the only varying factor is the local electric field due to the change of configuration of the system, while the conductance and length of the bridge are invariants. Therefore, the only determining parameter is $E^{\text {loc}}$. The larger is the local electric field, the larger is the current, resulting in a shorter charge transfer time and consequently resonance at a shorter wavelength.

**Figure 6:**
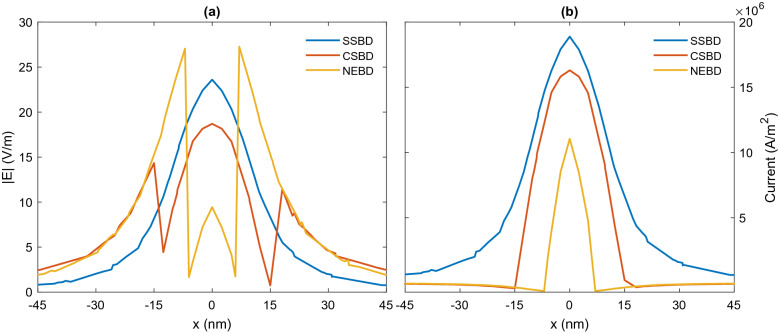
The variations of (**a**) |*E*| and (**b**) current along the monitoring lines across the gap areas for the bridged nanodimers at resonance.

To monitor the variations of the electric field intensities and current in the bridge, a series of auxiliary lines along the x-axis are used. The results shown in Fig. 6 denote that the strength of the $E^{\text {loc}}$ decreases as the configuration changes from SSBD to CSBD and NEBD, resulting in the reduction of current. As a consequence, the transfer time increases, and the plasmon mode of CTP experiences the redshift. SSBD and NEBD generate the highest and lowest local electric field inside the bridge, respectively, corresponding to the smallest and largest current flow across the junction. Note that although the CTP moves toward the longer wavelengths, the line width is broadened. The broadening of CTP can be inferred from the power dissipation in the bridge. The higher is the dissipation, the broader is the line width.

**Figure 7:**
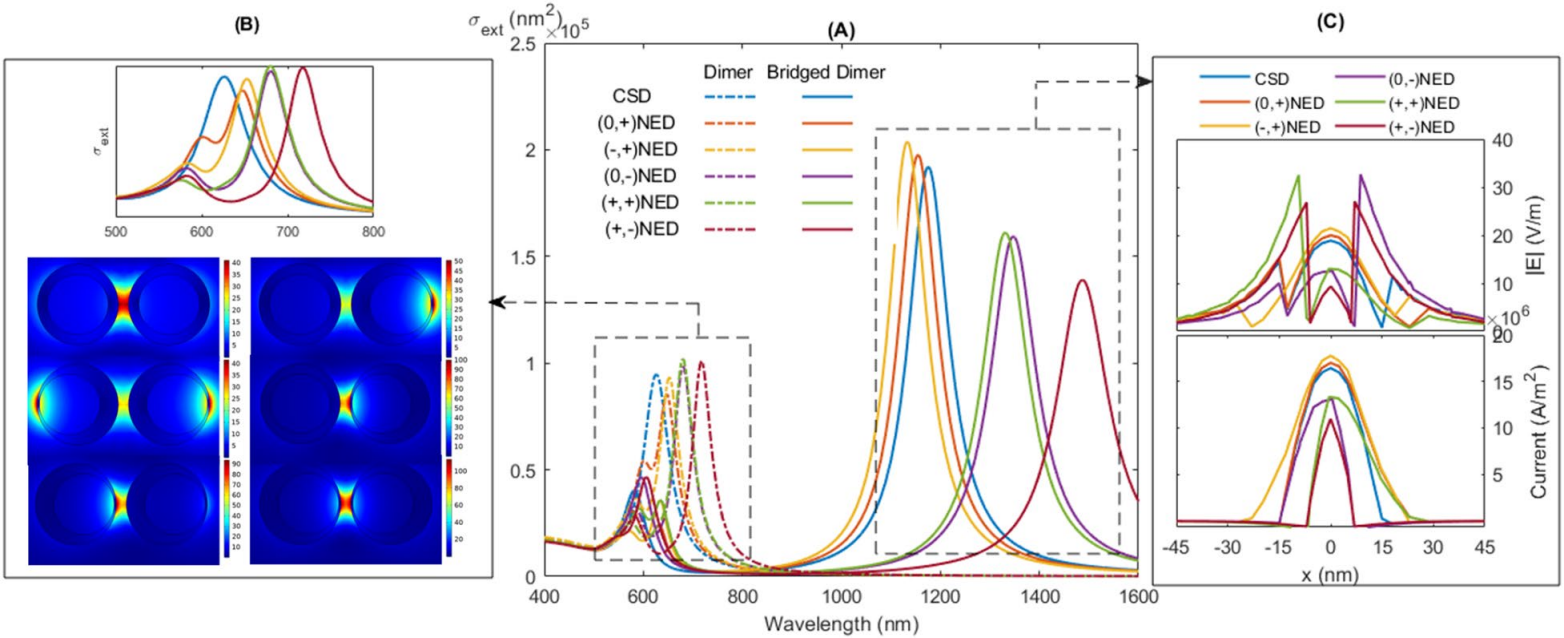
(**A**) Extinction spectra of dimers and bridged dimers composed of nanoeggs with different offsets, (**B**) E-field maps of different dimers calculated at the resonance wavelength, and (**C**) The variations of (UP) |E| and (Bottom) current along the monitoring lines across the gap areas for the bridged nanodimers at resonance.

As mentioned, two nanoeggs can place next to each other in different ways depending on the direction of their offset along the dimer axis. Therefore, the effect of orientation has also been explored to get better control over the optical spectra of dimers and bridged dimers. When the core moves to left/right, then the core offset parameter in the nanoshell becomes negative/positive. This nanoegg is called negative/positive nanoegg (NE−/NE+). Note that nanoegg with zero offsets is equivalent to core-shell. To investigate the dependence of plasmonic properties on the orientation, all possible compositions of two nanoeggs are considered including pairs of core-shell and positive NE ((0, +)NED and (0, +)NEBD), core-shell and negative NE ((0, −)NED and (0, −)NEBD), two positive nanoeggs ((+, +)NED, (+, +)NEBD), negative and positive nanoeggs ((−, +)NED, (−, +)NEBD) and positive and negative nanoeggs ((+, −)NED, (+, −)NEBD). The optical responses of these NED and NEBD are presented in Fig. 7.

From Fig. 7A, it is clear that introducing asymmetry leads to redshift of BDP in the extinction spectra. The BDP of CSD, (0, +)NED, (−, +)NED, (0, −)NED, (+, +)NED and (+, −)NED is redshifted from 625 nm to 648 nm, 653 nm, 680 nm, 681 nm and 719 nm, respectively. As mentioned, the higher hybridization strength between individual modes leads to larger redshift. The strength of hybridization and thus resonance displacement is determined by (1) degree of asymmetry and (2) the maxima of electric fields at the nanogap. Therefore, it is expected that the resonance peak of symmetric CSD is located at the blue side of the spectrum (shorter wavelength) relative to the peaks of asymmetric structures. To compare the resonance wavelengths of asymmetric geometries, the maxima of electric fields in nanogap must be considered. Figure 7B denotes that the highest E-filed maximum within the gap corresponds to (+, −)NED, followed by (+, +)NED , (0, −)NED, (−, +)NED and (0, +)NED. Therefore, the longest resonance wavelength corresponds to most asymmetric configuration of (+, −)NED with the highest E-field maximum in the nanogap. Note that although the electric field distributions of (0, +)NED and (−, +)NED do not show the maxima in the nanogap, the asymmetric geometries lead to slight redshift of resonance wavelength.

We address now in more detail the spectral features and tendencies of the CTP as optical fingerprints of conductive coupling. From Fig. 7A, the resonance of CTP for (−, +)NEBD, (0, +)NEBD, CSBD, (+, +)NEBD, (0, −)NEBD and (+, −)NEBD occurs at 1133 nm, 1156 nm, 1176 nm, 1332 nm, 1347 nm and 1487 nm, respectively. It is shown that the time needed for the charges to cross the junction determines the amount of the redshift of CTP. The larger is the local electric field, the larger is current, resulting in a shorter charge transfer time and consequently resonance at a shorter wavelength. Figure 7C shows the variations of the electric field intensities and the current in the bridge. It is clear that by increasing charge transfer time caused by decreasing the strength of the local electric field and the current across the junction, it is possible to tune the plasmon mode of CTP to the longer wavelengths.

**Figure 8:**
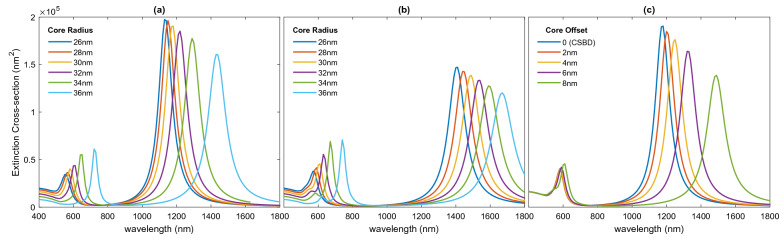
Effect of core size on extinction spectra of (**a**) CSBDs and (**b**) NEBDs. (**c**) Effect of core offset on extiction spectra of NEBDs.

It was found that the shape of nanoparticles has a dominant effect on the spectral feature of the bridged dimer. Compared to SSBD, dimers composed of two linked nanoshells show higher tunability by changing the ratio of core to shell radii or core offsets. Figure 8a,b illustrates the effect of core size on the optical response of bridged dimers, where the core radius increases from 26 to 34 nm and the radius of nanoshell is set to 40 nm. The results clearly indicate that increasing the core radius leads to a shift of SBDP and CTP peaks towards longer wavelengths. Moreover, increasing the core radius gives rise to a decrease in the CTP resonance intensity and an increase in the amplitude of SDBP. Note that the emergence of higher-order plasmon modes is only observed in the spectra of NEBDs with large core radii. When the core radius is small, the interaction between dipole-quadrupole modes is very weak, while the strength of this interaction is enhanced and a new resonance mode appears in the spectrum for NEBD with the larger core radius. Similarly, the effect of core offset on the spectra of NEBD is investigated by keeping the core and shell radii fixed at 40 nm and 30 nm, respectively, and changing the core offset from 2 to 8 nm. The results shown in Fig. 8c denote that there is a considerable redshift of CTP with a gradual decrease in its intensity as the core offset increases. The BDP and SDBP experience a slight redshift with an enhancement in their amplitudes.

### The role of junction

Alternatively, keeping the geometry of nanoparticles fixed, one can tune the spectral feature of the bridged dimer by controlling the conductance of the junction. According to Eq. (1), the conductance of the bridge can be enhanced either by increasing its contact area and conductivity or by decreasing its length. Therefore, the time of charge transport is decreased, resulting in the displacement of CTP modes toward the shorter wavelengths. Here, it is assumed that the conductivity of the bridge is fixed and it is similar in magnitude of gold. Consequently, the resonance wavelength of CTP easily can be tuned by changing the geometric parameters of the bridge.

**Figure 9:**
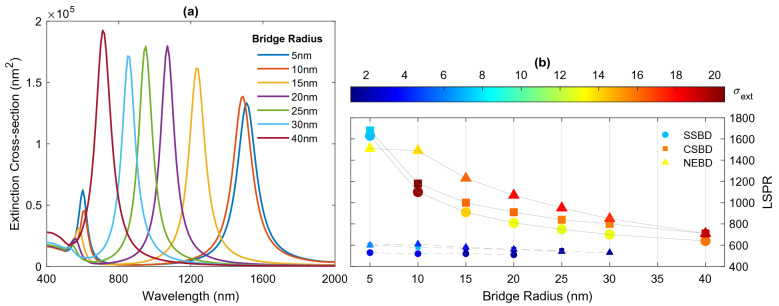
(**a**) Extinction spectra of NEBDs with different bridge radius. (**b**) Variation of resonance modes and their intensity by the radius of the bridge. Solid and dashed lines relate to CTP and SBDP modes, respectively.

Initially, by keeping the length of the junction fixed, the effect of the bridge radius on the spectral feature of the bridged dimers investigated. For a fixed junction length of $L=10 \; \text {nm}$, the radius of the bridge varies in the range of 5–40 nm. Figure 9a shows the extinction spectra of NEBD as a function of the radius bridge. On the one hand, when the width of the bridge becomes larger, the conductance is increased, resulting in the clear blueshift of CTP. On the other hand, the SBDP tends to disappear as the bridge radius increases. Finally, where the width of the bridge is equal to the diameter of the nanoparticles, the dimer behaves as one nanostructure with the shape of a nanorod. Therefore, only one resonance mode is observed in the spectrum, corresponding to the longitudinal plasmon mode of the large nanorod. Furthermore, the extinction efficiency of the resonance shows a considerable dependence on the bridge width. As the bridge radius increases, the amplitude of CTP mode is increased, while the strength of SBDP is decreased until it finally disappears from the spectrum. The CTP mode exhibit a blueshift from 1510 to 950 nm, as the bridge radius increases from 5 to 30 nm. With further radius increase, the nanoparticle acts as a continuous particle rather than a coupled pair of particles, consequently, the nature of the resonance mode is changed to the BDP mode at shorter wavelengths. for $a = 30\; \text {nm}$ and 40 nm, the resonance peaks of the nanostructures are located at 850 nm and 710 nm, respectively. Similarly, the effect of the bridge radius on the optical properties of SSBD and CSBD is also analyzed and its details are provided in Fig. 9b.

**Figure 10:**
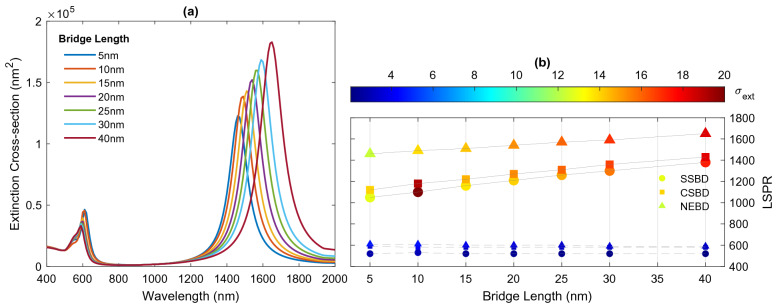
(**a**) Extinction spectra of NEBDs with different bridge length. (**b**) Variation of resonance modes and their intensity by the length of the bridge. Solid and dashed lines relate to CTP and SBDP modes, respectively.

The effect of bridge length on the properties of CTP is also investigated, where the radius of the bridge is kept constant at 10 nm, and the length of the bridge is increased from 5 to 40 nm. Figure 10a displays the corresponding extinction spectra of NEBD as a function of junction length. It appears that the CTP can be tuned from 1460 to 1650 nm quite simply, by increasing the bridge length and thus reducing the conductance of the junction. In other words, the increase in the bridge length leads to a redshift in the CTP due to weaker restoring force between the oscillating charges on two nanoparticles^59^. Furthermore, the intensity of CTP is monotonically increased with the junction length. In contrast, the effect of the bridge length on the bonding plasmon modes is insignificant and its wavelength and strength almost remain constant. Figure 10b displays the effect of junction length of the spectral features of SSBD and CSBD.

## Conclusion

In this paper, a detailed theoretical study of the optical response of capacitive and conductive coupled nanoparticles (i.e., dimer and bridged dimer) is numerically investigated. When the gap between the two nanoparticles is filled with a capacitive material, the optical spectrum exhibits a bonding dimer mode, while two different plasmon modes are identified in the optical spectra of two connected nanoparticles via a conductive bridge; high energy screened dipole plasmon mode and low energy charge transfer plasmon mode. It was found that such transition behavior is quite general, as similar effects can be obtained for dimers of different shapes: dimers composed of two spheres, core-shells and nanoeggs. However, the wavelength and intensity of the specific resonance modes are different. For capacitive coupling, the coupled nanoeggs achieve the strongest electric field in the gap at the bonding plasmon mode, followed by coupled core-shells and spheres. In the conductive situation, the shape of the nanoparticles affects the CTP modes significantly but affects the SBDP mode slightly. The coupled nanoeggs exhibit a most remarkable redshift of CTP, followed by core-shells and spheres. Furthermore, the effect of conductance of the junction is also investigated. When the conductance of the junction is increased, either by increasing the radius or decreasing the length of the bridge, the charge transfer plasmon mode is prominent in the extinction spectrum, while the spectrum is dominated by bonding dimer plasmon mode when the conductance becomes small. The results reveal that the impact of bridge radius on the spectral feature of linked nanoparticles is much greater than the effect of bridge length.
